# Personalized medicine in multiple sclerosis: hope or reality?

**DOI:** 10.1186/1741-7015-10-116

**Published:** 2012-10-04

**Authors:** Tobias Derfuss

**Affiliations:** 1 Department of Neurology, University Hospital Basel, Petersgraben 4, 4031 Basel, Switzerland; 2 Department of Biomedicine, University Hospital Basel, Hebelstrasse 20, 4031 Basel, Switzerland

**Keywords:** biomarker, multiple sclerosis, personalized medicine

## Abstract

Personalized treatment is highly desirable in multiple sclerosis because it is an immensely heterogeneous disease. This heterogeneity is seen in both the disease course and the treatment responses. Currently, a combination of clinical features and imaging parameters in magnetic resonance imaging is used to classify active and non-active patients and treatment responders and non-responders. Although this classification works on a group level, individual patients often behave differently from the group. Therefore additional biomarkers are needed to provide better indicators for prognosis and treatment response. Basic and clinical research have discovered different promising targets. It is now essential to verify the utility and accuracy of these markers in large, prospectively sampled patient cohorts.

## Review

### Background

Multiple sclerosis (MS) is a chronic autoimmune disease of the central nervous system. Because it hits patients early during life it has a major impact on a large part of their lives, and imposes a considerable economic burden. Current treatments for MS mainly target inflammatory processes, and there has been scant progress in treatments that enhance neuronal or glial regeneration. Therefore the current treatment strategy is to start treatment early to prevent neurodegeneration from the beginning. However, different problems arise when patients are treated after the first sign of the disease. First, does the patient need treatment at all, because he or she may have a benign disease course? Second, to which of the current baseline therapies will the patient respond best? Third, does the patient need an induction therapy? Fourth, is the patient at an increased risk for serious side effects?

Data from clinical trials provide information about efficacy and safety on a group level. However, it is obvious that treatment decisions in clinical practice must be made on an individual basis. This requires a personalized medicine approach. Biomarkers that could predict disease course, treatment response, and risk of side effects would be highly appreciated. Despite extensive research over the last years, few biomarkers have made their way into clinical practice. This mini review aims to summarize the state of current biomarker development in MS and promising new approaches.

### Predicting disease activity in multiple sclerosis

MS is a highly heterogeneous disease. This is probably not only true for the etiology, pathological features and autoantigenic targets but also for the disease course and response to treatment. Data from natural history cohorts show a broad spectrum of disease severities. The disease course is benign in 10% to 15% of patients and they do not need an assistive device for walking even after 20 years of MS [1]. On the other end of the spectrum, there are fulminant courses of MS that lead to severe disability within a few years. This heterogeneity of disease severity has obvious consequences for treatment decisions. In patients with a more aggressive disease, there is a need for early and aggressive treatment. Although this aggressive treatment poses some risks, these risks would be accepted by the patient and treating physician knowing that the benefits in this specific patient outweigh the potential risks.

Can we predict the disease course? There seem to be some clinical indicators that point to a worse disease course, such as high relapse rate during the first two to three years, bad recovery from relapses, and motor symptoms early during disease [2-4] (although these predictors have been disputed by others [5,6]). In addition, paraclinical tests such as high lesion load in magnetic resonance imaging (MRI), lipid-specific IgM oligoclonal bands and certain electrophysiological parameters hint at a more active disease (Table 1) [7-9]. However, while all these factors work fine on a group level, their predictive power in individual patients is low. Nevertheless, MRI is extensively used in clinical practice as a surrogate marker for the burden and activity of disease [10]. It can be expected that compound MRI measures that combine classical sequences like T2 and contrast enhanced T1 with more sophisticated measures like diffusion tensor imaging, double inversion recovery and magnetization transfer ratio will enhance the predictive power of MRI [11]. These techniques will allow for the detection of grey matter lesions that occur early during the disease course and that seem to correlate better with disability than white matter lesions that are detected with the standard T2-weighed imaging [12].

**Table 1:** Summary of established and potential biomarkers for diagnosis, prognosis, and treatment of multiple sclerosis.

Established biomarkers	Diagnosis	Prognosis	Treatment response/side effects
Cerebral spinal fluid-specific oligoclonal bands	+	(+)	-
Intrathecal immunoglobulin production	+	(+)	-
Intrathecal anti-viral immunoglobulin production	+	-	-
Magnetic resonance imaging	+	(+)	(+)
Neutralizing antibodies against beta-interferon	-	-	(+)
Neutralizing antibodies against natalizumab	-	-	+
Antibodies against JC virus	-	-	+
Aquaporin 4 antibodies	+	(+)	(+)
***Potential biomarkers***			
CD56 bright natural killer cells	-	-	(+)
Cytokines/chemokines	-	-	(+)
Myelin oligodendrocyte glycoprotein antibodies	(+)	-	-
Intrathecal/oligoclonal immunoglobulin M production	-	(+)	-
Transcriptomics	-	-	(+)
Genetics	(+)	(+)	(+)

+ valid biomarker that is used in clinical practice; (+) there is experimental and clinical evidence for this biomarker, but the need for more clinical data; - there is no clear evidence.

### How to pick the right medication for the right patient?

To date, choosing the right first-line therapy is based on guessing rather than knowing. On the one hand, one has to consider the activeness and severity of the disease when choosing a therapy. On the other hand, one has to take into account that no treatment has a responder rate of 100%. If a patient is placed on a treatment that does not work perfectly, this patient will lose precious time and will still have the risks of the treatment. In addition, society will have costs without benefit. Therefore there is an immense need to establish biomarkers that can predict treatment responses. So far, the response to a treatment is judged by counting clinical relapses, disability progression, and new lesions in MRI after one year of treatment [13]. Established biomarkers that are correlated with treatment responses include neutralizing antibodies against IFNs and natalizumab (Table 1) [14,15]. Genetic markers have not proven to be of use in predicting treatment response, so far. Recent studies on transcriptional profiles (both mRNA as well as miRNA) might reveal IFN response markers but this has to be reproduced in further longitudinal patient cohorts [16].

Another class of biomarkers is defined by the detection of pathologic immune responses, primarily antibody responses, against putative autoantigens [17]. These have been established as biomarkers in other neuroimmunological diseases such as myasthenia (antibodies against acetylcholine receptor) and paraneoplastic disorders (anti-Hu, anti-Yo and so on). In demyelinating diseases of the central nervous system, aquaporin 4 (AQP4) and myelin oligodendrocyte glycoprotein (MOG) have emerged as interesting antibody targets. Antibodies against AQP4 are associated with the clinical spectrum of neuromyelitis optica [18]. Because neuromyelitis optica seems to have a more aggressive course than general MS and seems to respond better to classical immunosuppression than to immunomodulation, detection of AQP4 antibodies helps to classify patients and aids in treatment decisions. Antibody responses against MOG are mainly found in pediatric demyelinating diseases like acute disseminated encephalomyelitis and pediatric MS [19]. MOG antibodies might help in differentiating between viral and autoimmune encephalitis [20]. Monophasic acute disseminated encephalomyelitis and pediatric MS might also be separated by MOG antibodies because these antibodies tend to persist longer in pediatric MS [19]. However, more longitudinal data are needed to corroborate this finding. If it is true, persisting anti-MOG antibodies would aid in guiding prophylactic treatment regimens. The role of MOG antibodies in adult MS is still speculative. More research is needed to clarify if MOG antibodies can be used for prognosis or classification of adult MS patients. A very recent discovery is the increased humoral immune response against KIR4.1 that was found in different cohorts of patients with MS compared with different control cohorts [21]. KIR4.1 is a rectifying potassium channel expressed by astrocytes and oligodendrocytes. Injection of KIR4.1 specific IgG (derived from MS sera) into the cisterna magna of mice caused pathological changes like complement deposition and loss of KIR4.1 antigen. As with MOG, it remains to be seen if the immune response against KIR4.1 will be useful as a biomarker for diagnosis, prognosis or treatment responses.

Another novel biomarker that may predict treatment responses early during therapy was discovered during clinical development of daclizumab, a monoclonal anti-CD25 antibody. It was shown that blocking the high-affinity IL-2 receptor (CD25) by daclizumab led to the expansion of a subtype of NK cells that show a high expression of CD56. This cell type seems to have immunoregulatory functions [22]. Expansion of CD56bright NK T-cells correlated with decreased MRI activity during daclizumab therapy in a phase 2 trial and may therefore indicate a patient population that preferentially responds to this treatment [23]. Despite these promising new avenues of research, we are currently left with clinical markers of treatment responses.

### Can we predict the risk of serious side effects?

When using immunosuppressive or immunomodulatory treatment, we are often confronted with serious side effects such as increased risk for infections. These risks often increase with the effectiveness of the treatments. A prominent example is the treatment with natalizumab, a monoclonal antibody against an integrin that inhibits migration of lymphocytes into the brain. This treatment shows an impressive reduction of relapses and disease activity in MRI [24]. The major drawback of this treatment is, however, the increased risk for a progressive multifocal leukoencephalopathy (PML) [25]. A careful review of PML cases in a post-marketing safety program revealed that a longer duration of therapy and previous immunosuppressive treatment are correlated with an increased risk of PML. As far as we know, PML is a reactivation of a preexisting latent infection with JC virus. A specific ELISA for the causative JC virus was developed that indicates if a patient harbors latent JC virus [26]. Using these three parameters (treatment duration, previous immunosuppressive treatment, JC virus antibody status), a risk stratification algorithm has been established that can be used in clinical practice to counsel patients that are on current natalizumab treatment or that are suited to go on the treatment [25].

Another biomarker that could predict autoimmune side effects of a treatment with alemtuzumab (monoclonal antibody against CD52 that depletes lymphocytes and monocytes) has been identified in early clinical studies. A surprising finding was made during the early clinical development of alemtuzumab: the occurrence of autoimmune thrombocytopenia and thyroid disease [27]. The pretreatment levels of IL-21 in serum correlated with the later development of autoimmune reactions [28]. Obviously, more prospective data are needed to confirm the value of this test in clinical practice.

The genetic background of a patient could also be used as a personalized biomarker. With the advent of high-throughput genetic screening approaches, genetic data are available in high quality and at ever decreasing costs. Some health authorities already demand genetic testing for certain human leukocyte antigen (HLA) haplotypes to predict the risk of serious cutaneous adverse effects of carbamazepine treatment [29]. In MS, an increasing number of genetic polymorphisms have been correlated with the disease [30] but so far their power to aid in diagnosing MS is low [31]. A large number of genes (including *GSTM*, *IL1B*, *PD-1*, *CCR5*, *OPN*, *IL4*, HLA-DRB1*1501, *CD24*, *ESR1*, *CD59*, *CNTF*, *CRYAB*, *IFNγ*, *MEFV*, *APOE*, *TGFB1*) have been associated with certain MS phenotypes but these correlations were often controversial [32].

Research on the pharmacogenomics of MS is increasing but a useful biomarker for clinical practice has so far not emerged [33]. Nevertheless, a recent study analyzing the functional consequences of a TNF receptor 1 polymorphism linked to MS shed light on possible reasons why a TNFα blockade failed as therapy in MS when they have been effective for other autoimmune diseases [34,35]. Gregory and colleagues [35] showed that this polymorphism leads to a novel soluble TNF receptor that can block TNFα, suggesting that a TNFα blockade in MS contributes to its pathogenesis rather than protecting from it. This study suggests that the functional analysis of genetic variants might help to predict autoimmune side effects related to specific immune pathways.

### Future directions and conclusions

The treatment armamentarium of MS has increased tremendously over the last few years and more treatments are close to registration. Although a final cure for MS is still missing, MS will be manageable in most patients with these treatments. The most important challenge regarding these therapeutic interventions will be to tailor the therapy to the needs of the patients and the aggressiveness of the disease. This asks for the development of biomarkers, either clinical, genetic, imaging or immunological, that allow for a better stratification of patients. An important prerequisite of biomarker development is the availability of longitudinal patient cohorts that are followed up over years. These cohorts could provide prospectively collected clinical information as well as genetic, imaging and immunological data. Many biomarkers have been proposed in clinical research. To finally bring them to clinical practice needs academic and industry co-operation. This joint effort will bring us one step closer from the hope to the reality of personalized medicine in MS.

## Abbreviations

AQP4: aquaporin 4; ELISA: enzyme-linked immunosorbent assay; IFN: interferon; HLA: human leukocyte antigen; Ig: immunoglobulin; IL: interleukin; miRNA: microRNA; MOG: myelin oligodendrocyte glycoprotein; MRI: magnetic resonance imaging; MS: multiple sclerosis; NK: natural killer; PML: progressive multifocal leukoencephalopathy; TNF: tumor necrosis factor.

## Competing interests

TD serves on scientific advisory boards for Novartis, Merck Serono, Mitsubishi Pharma, Biogen Idec, Teva and Bayer Schering Pharma; has received funding for travel and/or speaker honoraria from Biogen Idec, Novartis, Merck Serono, and Bayer Schering Pharma; and receives research support from Biogen Idec, Novartis, Merck Serono, the European Union, and the Swiss MS Society.

## Authors' information

TD is a clinical neurologist who specializes in neuroimmunology. He heads the outpatient and MS center at the Department of Neurology and is research professor at the Department of Biomedicine at the University in Basel. His research focuses on the discovery of new autoantigens, on biomarkers and on the analysis of the mode of action of disease-modifying treatments in MS. He is also involved in clinical trials for newly emerging therapies in MS.

## Acknowledgements

I'm grateful to Nicholas Sanderson for helpful comments.

## Pre-publication history

The pre-publication history for this paper can be accessed here:

http://www.biomedcentral.com/1741-7015/10/116/prepub
